# Status quo and problem analysis of cervical cancer screening program in China: Based on RE-AIM framework

**DOI:** 10.3389/fpubh.2022.987787

**Published:** 2022-10-14

**Authors:** Jingfen Zhu, Zhenghao Ge, Jiawei Xia, Qi Liu, Qingqing Ran, Yongbin Yang

**Affiliations:** ^1^School of Public Health, Shanghai Jiao Tong University, Shanghai, China; ^2^Department of Obstetrics and Gynecology, Shanghai General Hospital, Shanghai Jiao Tong University School of Medicine, Shanghai, China

**Keywords:** cervical cancer screening, health service program, RE-AIM framework, qualitative study, information system

## Abstract

**Background:**

An organized cervical cancer screening program is an effective method to prevent and control cervical cancer. This study aims to find barriers and facilitators in the implementation process of National Cervical Cancer Screening Program in Rural Areas (NACCSPRA) in China through program evaluation, and thus propose suggestions for optimization of the program.

**Methods:**

Through stratified sampling, 8 provinces (autonomous cities/districts) in eastern, southern, western, northern, and central China were selected for evaluation of NACCSPRA based on the RE-AIM framework. We obtained 15 program providers' experience and perspectives through semi-structured interviews. The data was analyzed using a combination of deductive and inductive analysis methods.

**Results:**

The study found that NACCSPRA mainly serves women with rural household registration or urban minimum living guarantee. Population mobility and certain demographic characteristics such as low education and poor health awareness are common participation barriers, while program publicity acts as a facilitator. A screening program's direct benefit is to promote early detection and treatment of cervical cancer, and its perceived indirect effect is to raise people's health awareness. The proportion of regions adopting the project is relatively high, and factors affecting employees' participation are screening workload, working environment, welfare benefits, degree of preference for grassroots work, and whether the project is included in the performance appraisal; In terms of implementation, there are disparities in screening methods, network informatization levels, and capital investment in various regions. Poor development of screening information system and insufficient screening funds are significant barriers to improvement of project implementation. In contrast, the overall implementation of follow-up is better; related policies issued by the local government and financial subsidies for poor women ensure the maintenance of the project.

**Conclusion:**

Shortage of funds is an important problem faced by current screening project, which negatively influences upgrade of cervical cancer prevention strategy, implementors' working environment, and impedes improvement of information network. In addition, defects in population coverage, especially in mobile population also deserves attention. The study found barriers and facilitators of NACCSPRA perceived by project providers and provided a theoretical foundation for project optimization.

## Introduction

Cervical cancer is the fourth most common cancer and cause of death among women worldwide. In 2020, 6,04,127 newly diagnosed cases and 3,41,831 death cases were reported worldwide (1). Significant regional differences in cervical cancer morbidity and mortality, which are associated with regional economic development, medical resource allocation, and the implementation of cervical cancer prevention and control projects, should not be neglected. In high-income countries, due to widespread vaccination, effective implementation of screening programs, improvement of cancer detection and treatment technologies, morbidity, and mortality of cervical cancer have dropped significantly. However, incidence still tends to increase in Eastern Europe and parts of sub-Saharan Africa (2). In 2020 WHO issued the global strategy to accelerate the elimination of cervical cancer through tertiary prevention, putting forward the target of reaching the goal of 90-70-90 by 2030.

Age-standardized incidence and mortality of cervical cancer in China in 2020 were 10.2 and 5.3 per 1,00,000 respectively, which shows that cervical cancer is still an important cause of disease burden (3). HPV vaccination and routine cervical cancer screening have proven to be effective in preventing cervical cancer (4). However, coverage of HPV vaccination is low in China because of the high price and limited supply of HPV vaccine. As a result, cervical cancer screening is now the primary method of prevention in China. In 2009, China launched the National Cervical Cancer Screening Program in Rural Areas (NACCSPRA) targeted at women aged 35–64 with rural household registration or who reached the standard of urban minimum living guarantee, series of testing including HPV testing (only conducted in HPV pilot areas), cervical cytology testing, colposcopy examination, and histopathological examination are conducted, only women with abnormal testing result receive next-level examination, and then final follow-up or treatment regimens are designed based on testing results. Nearly120 million free cervical cancer screenings have been carried out up to 2019 and the coverage rate of cervical cancer screening in China has increased to 21.4%, but it is still significantly lower than that in high-income countries and regions, such as Sweden (82%) (5) and America (81.6%) (6). Furthermore, prediction models have revealed that, under current prevention strategies in China, the incidence of cervical cancer still has the tendency to rise (7). This suggests that China's screening strategies and project implementation remain problematic and need to be improved.

The Reach, Effectiveness, Adoption, Implementation, and Maintenance (RE-AIM) framework was proposed by Russell E. Glasgow et al. It contains reach (R), effectiveness (E), and maintenance (M) dimensions at the individual level, as well as adoption (A), implementation (I), and maintenance (M) dimensions at staff and setting level, and definitions of each dimension are clarified in Glasgow's commentary (8). Since its inception, the model has been widely used in the systematic evaluation and planning of clinical research (8) as well as local and national health projects (9), including the evaluation of intervention effects on obesity, diabetes, and other diseases (10), as well as the effects of some health education projects for medical staff or patients (11).

RE-AIM framework has also been used in the field of cervical cancer screening. A study in Argentina used the framework to compare the effectiveness of programmatic HPV testing and cytology-based screening, verifying the feasibility of effective HPV testing programs in the real world in middle-income countries (12, 13). Mizota et al. applied RE-AIM framework to evaluate effectiveness of invitation materials using social marketing and behavioral economics approaches for improving screening rate of several cancers (14). The RE-AIM framework is systematic and comprehensive, focusing more on translating theoretical research into practical application than traditional evaluation theories. Therefore, evaluating a project from various components and dimensions, balancing internal and external effectiveness, and paying attention to contextualized problems during the dissemination and implementation process (9). Currently, researches on cervical cancer screening in China mainly emphasize the comparison of different screening strategies using certain health outcomes such as screening coverage, the detection rate of precancerous lesions, and the incidence and mortality of cervical cancer (7, 15, 16), while ignoring the impact of other factors related to implementation process, such as intervention environment, implementors' complement and attitude toward the project, the impact of the policy environment, etc. The RE-AIM framework was selected for this study to address the issue of limited evaluation indicators in previous studies, identify potential roadblocks to the transition of theoretical screening strategies to feasible programs in a real-world setting, and thus promote the optimization of current and future screening programs.

Early detection of cervical cancer plays a vital role in better treatment and prognosis of the disease. Due to the large population in China, the incidence number of cervical cancer accounts for nearly one-fifth of the total incidence number worldwide. Therefore, the effective implementation of cervical cancer screening in China is of great significance in reducing the incidence and mortality rate of cervical cancer worldwide. Our study selected the RE-AIM framework to evaluate the implementation of NACCSPRA in China, to discover barriers and facilitators through the experience and perceptions of project providers, and thus, provide a theoretical basis for optimization of organization and implementation of screening programs in the future.

## Method

### Participants and setting

First of all, we divided China's provinces, autonomous regions, and municipalities into five large areas according to geographical location: eastern, southern, western, northern, and central part. For the cervical cancer screening project evaluation, 1–3 provinces in each region were selected using stratified random sampling. The population distribution determined the number of regions selected in each area. Finally, Shanghai, Zhejiang Province, Jiangsu Province in the eastern region; Yunnan Province in the southern region; Ningxia Hui Autonomous Region in the western region; Liaoning Province in the northern area and Anhui Province, Hunan Province in the central region, totally 8 provinces (autonomous cities/districts) are selected.

Then we contacted providers of NACCSPRA in those selected regions for interviews. Inclusion criteria for respondents are participation in on-site cervical cancer screening or organization and management of local NACCSPRA over the past year. Finally, 3 staff in Provincial Maternity and Child Health Centers, 7 staff in the municipal and county-level Maternity and Child Health Centers, 5 medical staff of cooperative medical institutions, and a total of 15 persons were selected to be interviewed. All members participated voluntarily and anonymously.

### Data collection

Under the guidance of the RE-AIM framework, we have collected qualitative data about the implementation condition of NACCSPRA in different regions. The qualitative data sources include interviews with program providers and analysis of screening documents and data.

A semi-structured interview guide was developed based on the RE-AIM framework (17), previous evaluation research (12, 13), and expert recommendations (Table 1). We collected background information, implemented pilot surveys and revised interview outline according to experts' suggestion before formal interview. From August 2020 to May 2021, face-to-face or online interviews with 15 participants were conducted by 5 researchers, guided by the self-designed interview outline. All researchers had interview experience before and received relevant training. Each interview lasted about 40–60 min, the content of which was recorded by audio recording, and then transcribed verbatim and verified by independent transcribers to ensure that the research findings were based on the interviewee's statement rather than the researcher's existing knowledge or experience. Audio materials were destroyed after transcription to protect the participants' privacy.

**Table 1:** Interview guideline and key findings based on RE-AIM framework.

**RE-AIM elements**	**Descriptions**	**Findings**
Reach	• Who's the target population, and how about their participation situation? • What factors contribute to the participation/non-participation of the participants? • What might have been done to attract more of the target population to participate?	• Target population: women aged 35-64 with rural household registration or reached the request of urban minimum living guarantee, and most regions expand the scope of screening • The coverage rate of the task volume is higher than 80% • Non-participation of the floating population is severe problem • Propaganda is a facilitating factor for screening participation
Effectiveness	• What's the perceived positive/negative impact of the screening program on participants?	• Promote early diagnosis and treatment of cervical cancer and precancerous lesion, and thus improve patients' prognosis and quality of their lives • Psychological stress and influence on family relationships are potential negative impacts of detection of cervical cancer
Adoption	• What factors contributed to the organization and its individuals taking up the intervention?
• What factors affect the participation and enthusiasm of staff members in related organizations?	• The NACCSPRA covered more than 80% of counties (cities, districts) nationwide, and many regions interviewed have expanded coverage areas in recent years. • Collaboration level between medical institutions reflected in different regions varies a lot. • There are differences in working enthusiasm of the staff, which are influenced by factors such as workload, working environment, employee benefits, and whether screening work is included in performance appraisal.
Implementation	• How was the screening program implemented? What influenced the implementation of each part of the program?
• How about the cost and budget of the program?	• Screening methods among different regions vary from Pap test and TCT to combined screening (HPV + cytological test). • The construction of information systems is inadequate and uneven in different regions, which can be roughly divided into three development stages. • The completion rate of follow-up is required to be higher than 95%, and most patients have high compliance. • The amounts of subsidies varies from place to place, and all regions reported an insufficiency in screening funding.
Maintenance	• What has been made to ensure the maintenance of the program?	• Different regions constantly adjust and improve relevant policies based on national projects and local situations. Some regions have also carried out regional-characteristic screening projects and issued subsidy funds to ensure those with positive screening outcomes receive appropriate follow-up treatment.

At the same time, we asked participants to provide relevant documents (policy documents, implementation scheme, etc.) and available data about participation of local cervical cancer screening projects if possible. Researchers sorted out valid information in those documents based on the RE-AIM framework as supplements to interview data and compared requirements in those documents with the actual implementation situation reflected by the interviewers.

### Data analysis

Within 24 h after the interview, the audio recordings were transcribed verbatim by an independent transcriber and saved in Microsoft Word documents separately. The researcher perused and be familiar with the content of the interviews. The data was then analyzed using a combination of methods, the first of which was iterative deductive content analysis. We take the RE-AIM framework as the initial coding structure, coding related contents by pre-designed codes according to 5 dimensions of the RE-AIM framework. Second, inductive content analysis was used to identify emerging codes and concepts that did not match the pre-designed codes, then sorting all codes into 5 dimensions of the RE-AIM framework and charting data into the framework matrix. During the data analysis process, research members hold a formal meeting every 2 weeks to discuss emerging codes in each dimension of the RE-AIM framework, their categorization, and interpretation, focusing on issues or disagreements generated to reach consensus.

## Result

### Reach

Reach, the criteria of degree of individual participation aim to study the population participation, representativeness of participants and non-participants, and possible influencing factors of the program's accessibility.

#### Target population

The target population of NACCSPRA is women aged 35–64 with rural household registration or who reached the standard of urban minimum living guarantee. Most regions expand the scope of screening, such as extending the covered age range to 70 years old, taking into women with certain conditions such as retirement, women in public welfare positions, women in single-parent families, etc. Women with urban household registration are also integrated into the target population in some pioneer districts.

All regions reach a coverage rate higher than 80% of the task volume. Those who aren't covered are screened primarily through hospital opportunistic screening and occupational health examinations, with no guarantee of participation.

“*Screening in the outpatient department isn't covered by health insurance, so acceptance depends on one's economic status, individual health awareness, and attention, and those out of coverage range rarely participate in screening initiatively.”*—Changsha Province

Non-participation has improved in recent years, but it has not completely disappeared. The mobile population is an important characteristic for non-participants, which was mentioned repeatedly by interviewees, as it requires household registration for a free screening. Among them, screening leakage of inflow population in economically developed areas and outflow population in underdeveloped areas are serious. Other general characteristics of non-participants include older age, lower education level, poor health awareness, and distrust of the medical system. In addition, cognitive biases on screening and the low family status of women will also affect participation, which is more serious in remote areas.

“*Screening barriers differ in different age groups. Many people under 45 work outside as migrant workers; some of those over 45 years old have a deviation in their understanding of screening, believing that screening is harmful to them or bad things, such as divorce, will happen if diagnosed with the disease.”*—Ningxia Province

#### Publicity of the program

Although respondents believe that propaganda facilitates screening participation, they also believe that common obstacles such as insufficient publicity, a lack of professionalism among personnel, and a lack of standardization in the form, frequency, effectiveness, and funding of publicity still exist. In addition, region-specific barriers should not be ignored, especially in remote regions, such as language barriers and inconvenient transportation.

“*Due to the complex terrain and scattered villages, residents do not have convenient transportation tools, sometimes even ambulances are used to pick up residents for screening. Another problem is that some people speak minority languages which propaganda staff cannot grasp, leading to communication barriers.”*—Yunnan Province

### Effectiveness

Our research mainly discusses the effectiveness of the program from the project providers' perspective. We focus on both positive and negative effects perceived by program providers and the reasons behind them.

Program implementers generally believe that the screening program is of great significance to promote screening coverage and early diagnosis and treatment of cervical cancer and thus reduce incidence. Besides the direct benefits, regular publicity can improve people's health awareness, and early detection of cervical lesions has a positive effect on a patient's quality of life. In contrast, the possibility of disease detection causing psychological stress or affecting family relationships is a potential negative impact.

“*Every year, it is possible to detect 2–3 cancers and 20–30 precancerous lesions. It is reasonable to say that it will reduce the incidence rate and improve people's awareness of the importance of screening.”*—Yunnan Province

“*Patients suffer from psychological stress during early treatment, such as anxiety and fear, but quality of life will be improved after receiving treatment, and corresponding lifestyle changes will be adopted by patients.”*—Ningxia Province

### Adoption

Adoption refers to the proportion and representativeness of the setting in which a given policy is adopted and the staff who participate in the program.

#### Covered area

The NACCSPRA covered more than 80% of counties (cities, districts) nationwide from 2009 to 2019. There were 21 provincial administrative regions that covered all rural areas and 13 that covered both urban and rural areas.

In recent years, many places interviewed have expanded screening coverage areas. For example, Ningxia Hui Autonomous Region has expanded from 6 counties (districts) to 22 counties (districts) and the Ningdong region since 2012; Dalian City achieved full coverage of agricultural counties in 2017.

#### Implementation institution

Screening tasks are assigned to primary screening institutions and receiving institutions. The former is responsible for initial screening and abnormal case management, and the latter should be capable of further diagnosis and treatment for those with abnormal screening results. The level of collaboration between medical institutions reflected varies among different regions. Shanghai, Zhejiang, and Yunnan reported insufficient information exchange among institutions and unclear distribution of responsibilities. In contrast, Ningxia and Anhui Provinces stated that the division of labor is clear with better cooperation.

“*Local maternal and child health care hospitals led the screening, and Women's Federation arranged staffs to attend classes, but participation level of other institutions was not high”*—Yunnan Province

Women are encouraged to go to primary screening institutions on their own or in groups in most areas. However, due to traffic problems and low screening awareness in Yunnan, screening teams are sent to the countryside for testing. Nevertheless, this method is limited in application due to time-consuming and laborious.

“*In some areas, we send screening teams to the countryside, but this method has problems of 10 more people needed, higher expense and poor site conditions”*—Yunnan Province

#### Attitude of program provider

After training and assessment, most screening staff are hired directly by a related screening agency. Although having a high degree of recognition of the project, heavy workload, noisy working environment, and poor welfare benefits negatively affect staffs' participation enthusiasm. Meanwhile, affection for grassroots work, including screening work into performance appraisal are stimulus factors. However, some reported that performance appraisal is mainly for screening institutions instead of individual physicians.

“*The screening workload is heavy, and sometimes extra work is inevitable. Each doctor screens more than 60 people every day with low salary, which may lead to complaints.”*—Shanghai

### Implementation

This dimension focus on intervention agents' fidelity to screening protocol, adaptations made to the original plan, and the cost of the intervention.

#### Screening method

Pap smears are used in Shanghai Pudong, Yunnan, and part of Anhui Province, TCT is used in Shanghai Fengxian District, Dalian Pulandian District, and Changhai County. Hunan, Ningxia, Zhejiang Province, and some HPV test pilot areas switched to combined methods of HPV and cytology for preliminary screening. Participants with abnormal preliminary screening results will be examined by colposcopy.

According to the findings, on-the-spot monitoring and sample checking are the most common methods of quality control. While regulations about quality control are contained in screening documents, a complete quality control implementation system remains absent.

“*The screening team will monitor screening quality on-site, and the Maternal and Child Health Center will select test results randomly for quality evaluation.”*—Shanghai

“*There are quality control requirements for sample testing, but conduction of regular spot checks will meet difficulties. Instead, we inspect screening work on-site for quality control.”*—Ningxia Province

#### Information collection and utilization

At present, patients diagnosed with cervical precancerous lesions or cervical cancer will be reported to the Regional Maternal and Child Health Information System and National Cancer Registry. But the development of screening information systems varies from place to place, of which the construction level can be roughly divided into 3 stages: The first stage, no screening information system (Shanghai Pudong District) or the system is still under construction (Ningxia). Participants' data collection is currently limited to on-site registration on paper. The second stage involves the establishment of regional registration systems to digitally input participant information, but the system is only accessible to staff and does not allow patients to interact (Liaoning). In the third stage, the information system is complete and doctor-patient interaction is achievable. Participants can use the system to inquire about their screening results, and online follow-up management is also available. For example, the “Artificial Intelligence Internet + Cloud Platform” in Yunnan Province and the “Healthy People's Livelihood Information System” in Changsha City have achieved remarkable achievements.

The construction of a large-scale and interchangeable information system has several obstacles to be resolved, such as high capital investment, difficulty in system operation, and using barriers for grassroots staff.

“*The biggest problem is insufficient funds. Although there is financial support from both national and provincial levels, it is very expensive to establish and maintain a relatively extensive information system. On the other hand, various obstacles will appear during the actual use process.”*—Ningxia Province

#### Follow-up

Follow-ups are carried out according to national requirements with corresponding follow-up strategies for different lesion grades, and follow-up rates are required to be higher than 95%. The completion rate increased through effective measures such as door-to-door notification of incompliant people. However, it's difficult to avoid isolated cases going unnoticed or refusing to take action.

“*Follow-up should last for at least 3 months and must have a final follow-up result. There used to be cases of loss to follow-up (such as changing the phone number), but now such things happen less often.”*—Anhui Province

“*Some participants will not strictly follow the recommended standards to receive a subsequent examination and corresponding supervision is deficient.”*—Zhejiang Province

The screening agency will make treatment recommendations for patients with varying degrees of disease severity. However, poor economic conditions, particularly in underdeveloped areas, are deterrents to patients continuing their treatment. In contrast, supports from medical staff and family members both have positive impacts.

“*Most patients are active for further treatment, and Women and Children Health Center will offer 20% discount on surgical treatment for those with positive screening outcome.”*—Liaoning Province

#### Cost

China's national two-cancer screening program stipulates that the standard of cervical cancer screening subsidy should be no < 49.6 yuan per person. An insufficient part of funds will be supplied by provincial, municipal, and county financial departments in a certain proportion; besides, the HPV pilot project has additional subsidies. The actual screening funds in various regions are composed of subsidies provided by the government and self-raised by appointing medical institutions. The number of subsidies varies from place to place ranging from 49.6 to 69 yuan/person except for Shanghai, where screening funding is higher than in other regions due to an additional 227.6 yuan/person (including funds for breast cancer screening) provided by the municipal government for the regional “two-disease screening project”.

All regions reported an insufficiency in screening funding, mainly caused by the following reasons: Firstly, cervical cancer screening subsidies are based on the cost of screening technology, leaving out funds for publicity, personnel, and equipment upgrades. Secondly, cervical cancer subsidies are distributed based on the task volume reported on the ground survey; however, the actual number of people who participate in the screening often exceeds the target number. Lastly, in several places, the screening method must be updated, resulting in an increase in screening costs, but the subsidy remains unchanged.

### Maintenance

Maintenance refers to the persistence of the program after some time. We mainly focus on the persistence at the setting level and the degree of institutionalization of the project.

Since the launch of the cervical cancer screening project, local governments have introduced relevant policies based on national requests and local economic and medical conditions, and continuously adjusted and improved them. Simultaneously, a special fund was established to ensure that poor women continued to participate in examinations and treatments.

“*Both provincial and county levels have their policy documents adjusted according to actual local conditions. The working mechanism is relatively flexible.”*—Anhui Province

“*There are very few cases that patients can't afford the medical expenses, because cervical cancer is considered as a major disease and large part of treating expense will be reimbursed by national insurance.”*—Shanghai

However, some interviewees claimed that the screening documents were not updated promptly or that the targeted age group of NACCSPRA was inconsistent with those required by the regional project such as Shanghai's “two diseases” (gynecological diseases and breast diseases) program, which will confuse some implementors.

“*The documents have not been adjusted since 2015, and there are contradictions between screening documents and updated guidelines. For example, experts advocate HPV as the first choice for cervical cancer screening, but it will bring an economic burden if HPV is added. In addition, Shanghai's “two diseases project” requested women under 70 years old to be screened, however, the two-cancer screening only requires women under 64 years old to be screened, so how to deal with the 64–70 age group?”*—Shanghai

## Discussion

We select representative areas in China and use the RE-AIM framework to conduct a comprehensive process evaluation of the NACCSPRA, exploring barriers and facilitators during the implementation process. Cost is an important focus of the dimension Implementation, and multiple areas reported a shortage of screening funds. Insufficient investment in the prevention and control of cervical cancer (18) is related to small screening coverage of women of the right year, deficiency in medical equipment and infrastructure, difficulties in construction of large-scale information systems, and inability to promote HPV vaccines (19, 20). Also, it was reported in various regions that poor welfare of implementation staffs caused by insufficient funding has undermined staffs' working enthusiasm, which may affect screening quality. Capital is of vital importance for the successful implementation of a project, however, it is unavoidable that only limited funding and resources can be invested into one project, as a result, the strategy for funding usage plays a crucial role. According to studies, population coverage is more important than the frequency of screening, so even with the current budget China can reach the elimination goal earlier by reducing the frequency of screening in exchange for extending screening coverage and increasing HPV vaccine coverage at the same time (7). In addition, repeated tests caused by free screening projects implemented along with unrestricted opportunistic screening might waste social resources and bring a greater financial burden (21, 22). As a result, reducing the number of repeated tests and developing a better screening strategy can alleviate the shortage of funds while also improving the prevention effect.

High adoption was reported at the setting level, government report shows that cervical cancer screenings currently covered 2,644 counties (cities, districts) in China, accounting for 87% of the total. There are differences in the degree of cooperation among medical institutions among different regions. Perceived cooperation levels may not be accurate because they are reflections with no objective evaluation standard. However, further clarification of responsibilities, improved inter-agency communication, and the establishment of an accountability mechanism are all essential. At the staff level, working activity can be stimulated by improving working benefits and environment, and establishing individual performance appraisals.

However, while the proportion of regions that adopted the policy is high, the screening coverage rate is still not high in China. The possible reasons are as follows: Firstly, the main population reached by the project is limited to women with rural household registration or subsistence allowances. Women not covered mainly screened by occupational physical examination and hospital opportunistic testing, however, outpatient screening services are not covered by medical insurance in some regions due to different medical insurance policies across China, which may negatively affect screening coverage rate. Considering that rural women have a relatively lower willingness to receive screening (23) and a higher HPV infection rate (24), under limited medical resources, this strategy for coverage is reasonable. Nevertheless, when compared with the target population in high-income countries such as Australia and Sweden, coverage of NACCSPRA in China is insufficient. And the study has shown that organized programs targeting a small portion of women cannot effectively reduce medical inequality (21, 22). Therefore, China must carry out a wider-scale screening project to reach the goal of 70% of women screened set up by WHO. Secondly, the screening program is not able to reach the entire target population. Deficient management of the floating population is an important factor for screening leakage. The mobile population's willingness to participate will be reduced as they have to pay for screening or return to their registered residence to receive free screening in most areas (25). Special management methods for the mobile population should be developed so that eligible women can be screened in current residence instead of registered ones. In addition, consistent with other studies, specific demographic characteristics and insufficient knowledge and awareness of cervical cancer also affect participation, especially in remote areas (23, 26–29), demonstrating the importance of paying attention to regional differences and population characteristics during project publicity as well as increasing the intensity and professionalism of project publicity (30, 31).

When discussing about the dimension of effectiveness, positive impact of the program was widely acknowledged by providers, which was consistent with previous studies (32). Although there may be negative effects on patients such as psychological pressure and stigma, the overall benefits far outweigh the disadvantages, early diagnosis and treatment have a positive impact on the long-term quality of life of patients.

Among different perspectives of implementation, there are disparities in screening methods, information network level, and capital investment in various regions. Cost and effect must be carefully considered when choosing screening methods. We found that most areas in China are transitioning from pap smear tests to TCT, but only HPV test pilot areas are conducting joint screening methods (HPV test+ cytological test). HrHPV testing has proven to be an effective primary screening method with high sensitivity (33–35), Netherlands, Turkey, and some other countries have already implemented HPV testing nationwide (36). Taking into account differences in regional development, China can adopt a diversified screening strategy and gradually transition to joint screening. HPV testing combined with cytological testing applies to economically developed areas (37); areas with poor resources can continue to use pap smear tests or apply AI-assisted TS technology (38, 39) and HPV self-sampling (40–42).

A comprehensive information system is critical for preventing screening leakage or repetition, improving screening results utilization, and improving inter-institutional cooperation efficiency. Currently, the cancer registry system in China is relatively complete, but the screening information system undergoes insufficient and uneven development among different regions. The main problems with screening information systems in China are as follows: the first one is the limited scale of information systems and the unavailability of information exchange between systems; the second is that information registration in most areas is limited to positive screening results, but previous screenings records (including screening time and results), HPV vaccination status are not contained. The third is the lack of interactivity of information systems at both hospital and patient levels. Both doctors and patients themselves cannot retrieve their previous screening records through the system. However, in the high-income countries, a unified electronic screening registration system and complete follow-up services have normalized (43, 44). Specialized applications for cervical cancer screening projects are being continuously developed (45, 46). This is especially important in China, where regional disparities and a lack of physician resources persist and suggests that the development of a national-wide information platform should be accelerated to achieve the goal of informatization of each process and establish an interactive “patient-platform-hospital” connection.

Although the completion rate of follow-up is high, attention should still be paid to individuals lost to follow-up. Wang et al. showed that the highest missing rate occurs during the process of calling back cytology-positive individuals to participate in colposcopy examination, leading to the loss of high-risk patients (47), which may cause the adverse outcome of disease (48). Possible reasons for loss of follow-up are missing data, diagnostic errors, and substandard quality control (47). The establishment of information systems with interactive functions can promptly remind individuals with positive screening outcomes to check the result and participate in subsequent diagnosis and treatment and make it possible for high-standard quality control and data inspection.

Maintenance of the screening program are ensured by national policy and regional characteristic projects, local departments implement the project in accordance with the policy documents issued by the National Health and Family Planning Commission and the National Women's Federation. At the setting level, timely adaption is important for sustainability of the program, so it is necessary to ensure timely adjustment of screening plan documents to make them match with developed regional screening capacity, changed expert guidelines, and the latest research progress on cervical cancer prevention and control. For example, Shanghai implemented regional characteristic “two diseases” program, which expanded screening disease categories and expanded age range of target population to 70 years old. However, the two programs are often combined in practice due to the overlapping of most examinations, making implementors confused about age range of target group to notify. Therefore, for better sustainability of the program, not only adaption is needed, but highlighting and clearly clarifying any adaption made during intervention to implementors is also indispensable. At the same time, to maintain individual benefits, it is necessary to construct convenient information access. Some countries publish screening information on a unified website timely, for example, the American Society of Colposcopy and Cervical Pathology (ASCCP) (49) simultaneously updates the latest screening information on both web pages and special applications. China can also summarize useful information about cervical cancer and screening programs on a specific website for participants to check in time, thus increasing the initiative of participation. On the other hand, maintaining individual benefits requires improving the ability for subsequent diagnosis and treatment, as well as providing financial assistance to women with financial difficulties.

## Strength and limitation

Currently, the RE-AIM framework is only used in a few studies to evaluate cervical cancer screening programs worldwide, and most of them are quantitative studies (12, 13). The importance of qualitative research, on the other hand, cannot be overlooked when using the RE-AIM framework (17). This study is the first qualitative study using the RE-AIM framework to evaluate cervical cancer screening projects in China, digging into why and how evaluation results were generated through the experience and opinions of project providers. On another hand, semi-structured interviews allowed exploration of unintended results, discovering barriers and facilitators in project implementation through providers' rich and contextualized expressions.

There are also limitations in our study. Firstly, due to the research scale's limitations, only 8 provinces or cities (regions) in China were selected for this study. But uneven regional development in China and disparities in screening coverage between regions (23) were taken into account during sampling by selecting regions in the divided eastern, southern, western, northern, and middle areas in China respectively as research objects. The representativeness of research objects makes up for the small sample size to a certain extent. Secondly, in our research, we discovered a variety of implementation barriers. Nevertheless, due to limitations of the RE-AIM framework itself, we cannot accurately assess the precise impact of each dimension on the screening effect, and further quantitative research is needed. Thirdly, this study used province as a sampling unit. However, during the interview, we found there may be differences in project implementation among different districts even in the same province. We suggest further research in representative regions using smaller administrative divisions as sampling units for more precise results.

## Conclusion

The study found barriers and facilitators of NACCSPRA perceived by projects providers. Our study found that shortage of funds is an important problem faced by current screening project, which negatively influences the upgrade of screening strategy, implementors' working environment, etc., thus affecting the screening effect. At the same time, the financial problem also hinders the scale-up and completion of information systems and improvement of interactivity, which reduces the efficiency of screening. In addition, defects in population coverage and allocation of financial resources also deserve attention. In order to achieve optimal resource allocation and screening effectiveness under limited resources, future research on the cost-effectiveness of screening implementation plans in contextualized settings will be required.

## Data availability statement

The datasets generated for this study are not publicly available due to concerns for participants' privacy, but are available on reasonable request to the corresponding author.

## Ethics statement

Ethical review and approval was not required for the study on human participants in accordance with the local legislation and institutional requirements. Written informed consent for participation was not required for this study in accordance with the national legislation and the institutional requirements.

## Author contributions

YY and JZ conceived and designed the study and revised the manuscript. JZ, ZG, JX, QL, and QR collected the data. ZG and JX analyzed and interpreted the data. JZ and ZG drafted the manuscript. All authors have read and approved the final manuscript.

## Funding

This work was funded by Shanghai Jiao Tong University Liberal Arts Scientific Research Innovation Cultivation Program (WKCX1930).

## Conflict of interest

The authors declare that the research was conducted in the absence of any commercial or financial relationships that could be construed as a potential conflict of interest.

## Publisher's note

All claims expressed in this article are solely those of the authors and do not necessarily represent those of their affiliated organizations, or those of the publisher, the editors and the reviewers. Any product that may be evaluated in this article, or claim that may be made by its manufacturer, is not guaranteed or endorsed by the publisher.
